# Patients’ perspectives on their motivations for participating in non-clinical medical teaching and what they gain from their experience: a qualitative study informed by critical theory

**DOI:** 10.1007/s10459-023-10262-7

**Published:** 2023-06-29

**Authors:** Julie Massé, Sophie Grignon, Luc Vigneault, Geneviève Olivier-D’Avignon, Marie-Claude Tremblay

**Affiliations:** 1 Faculty of medicine, Université Laval, 1050 avenue de la Médecine, Quebec City, Québec Canada; 2 Faculty of nursing, Université Laval, 1050 avenue de la Médecine, Quebec City, Québec Canada; 3 Vitam, Centre de recherche en santé durable, 2480 chemin de la Canardière, Quebec City, Québec Canada; 4 Patient-Partner, Quebec City, Québec Canada

**Keywords:** Patient engagement, Patient participation, Patient experience, Medical Education, Teaching and learning, Qualitative research

## Abstract

In 2019–2021, we engaged in a project aimed at developing, implementing, and evaluating an educational intervention actively involving patient-teachers in undergraduate medical education at Université Laval, Quebec, Canada. Patient-teachers were invited to participate in small group discussion workshops during which medical students deliberate on legal, ethical, and moral issues arising from medical practice. Patients were expected to bring other perspectives, based on their experience with illness and the healthcare system. Little is still known about patients’ perspectives on their participation experience in such context. Informed by critical theory, our qualitative study aims to document,: (i) the motivating factors for patients’ participation in our intervention; and (ii) what patients gained from the experience. Data collection was based on 10 semi-structured interviews with patient-teachers. A thematic analysis was conducted using NVivo software. Motivators for participation arose from: (i) perceived consistency between patients’ individual characteristics and those of the project, and (ii) conceiving the project as a means to reach individual and social goals. What patients gained mainly refers to (1) the appreciation of a positive, enriching, motivating yet uncomfortable and destabilizing experience; (2) a deconstruction of biases against the medical field and critical thinking about their own experience; (3) new knowledge, with a potential impact on their future interactions with the healthcare system. Results reveal patients as non-neutral thinking and knowing subjects, engaged in the participation experience as active teachers and learners. They also highlight the empowering and emancipatory nature of the learning gained through patients’ participation experience. These conclusions prompt us to promote transformative interventional approaches that question the pervasive power issues in medical teaching and value patients’ specific knowledge in teaching and learning the Art of Medicine.

## Introduction

In recent decades, there has been a paradigm shift towards partnership approaches with patients at different levels of the healthcare system (Pomey et al., 2015). This new paradigm, often known as ‘patient engagement’, has its roots in the democratic ideals of participation. It recognizes the relevance and value of patients’ experiential knowledge (Tuckett et al., 1985) and requires patients to have an active and central role within their personal healthcare team. This paradigm also involves revising health professionals’ training models towards a more active role for patients (Flora et al., 2016) beyond the instrumental roles historically assigned to them (Spencer et al., 2000; Jha et al., 2009; Towle et al., 2010; Towle & Godolphin, 2011).

According to the literature, engaging patients in medical teaching has the potential to contribute to the acquisition of key competencies in future physicians (Wykurz & Kelly, 2002; Jha et al., 2009; Towle et al., 2010; Massé et al., 2023). For instance, studies report benefits in terms of positive attitudes towards patients, knowledge and skills in communication and psychosocial fields. Moreover, patient engagement is increasingly seen as a promising avenue for promoting reflexive learning in medical students (Jha et al., 2009; Towle et al., 2010; Massé et al., 2023). The value added to student learning justifies the growing interest of the medical education research community in patient engagement practices. However, while most studies focus on associated student learning outcomes, few have addressed patients’ views of their own participation experience (Wykurz & Kelly, 2002; Spencer, 2010; Towle et al., 2010; Watts et al., 2015; Flood et al., 2018; Roebotham et al., 2018; Kline et al., 2022). Furthermore, most studies interested in the experiences of patients involved in medical teaching have focused on the presence of students in clinical consultations or in ward rounds, rather than on specific patient engagement educational programs or interventions (Watts et al., 2015).

### Review of the literature

Our review of the literature allowed us to identify a number of studies that explored patients’ participation experiences in hospital-based medical education (Kuan & O’Donnell, 2007; Ben Salah et al., 2015; Rockey et al., 2020) or in primary care and community clinical settings (Prislin et al., 2001; Coleman & Murray, 2002; Walters et al., 2003; Chipp et al., 2004; Choudhury et al., 2006; Haffling & Hakansson, 2008; Heathcote, 2008; Ezra et al., 2009; Hudson et al., 2010; Mol et al., 2011; Lucas & Pearson, 2012; McLachlan et al., 2012; Alao et al., 2021; Kjaer et al., 2021). These studies highlight that patients overall consider their experience of participating in clinical medical education positive or neutral (Prislin et al., 2001; Coleman & Murray, 2002; Walters et al., 2003; Choudhury et al., 2006; Kuan & O’Donnell, 2007; Haffling & Hakansson, 2008; Ezra et al., 2009; Hudson et al., 2010; Mol et al., 2011; McLachlan et al., 2012; Ben Salah et al., 2015; Rockey et al., 2020; Alao et al., 2021). In particular, McLachlan et al.’s phenomenological study (2012, p. 969) points out how patients perceive the presence of students in ambulatory consultations “had little potential to do harm, might be of minor benefit to patients, but was assumed to be beneficial to students and therefore to society”. Consistently, three main categories of factors motivating patients to engage in medical education in such clinical contexts emerge: (1) a sense of duty and obligation, interactions with real patients being considered normal and necessary for medical education (Chipp et al., 2004; Heathcote, 2008; McLachlan et al., 2012; Rockey et al., 2020), (2) altruistic considerations (i.e., to help future doctors and future patients and to give back to the healthcare system) (Coleman & Murray, 2002; Chipp et al., 2004; Heathcote, 2008; Rockey et a., 2020; Kjaer et al., 2021) and (3) the search for personal gain (e.g., better care, improved health knowledge) (Coleman & Murray, 2002; Chipp et al., 2004; Heathcote, 2008; Alao et al., 2021; Kjaer et al., 2021). The studies also highlight two broad categories of personal gains that patients consider they derive from their participation experience: (1) pragmatic, utility-maximizing gains (e.g., perceived improvement in quality of care in terms of medical expertise, consultation time and patient education, increased clinical knowledge about their health condition) (Prislin et al., 2001; Ezra et al., 2009; Mol et al., 2011; Lucas & Pearson, 2012; McLachlan et al., 2012; Alao et al., 2021) and (2) therapeutic gains (e.g., personal satisfaction; enhanced self-esteem and empowerment, sense of meaning and usefulness, enhanced happiness and well-being through the care pathway, new insights into their illness and care experience) (Walters et al., 2003; Haffling & Hakansson, 2008; Ezra et al., 2009; Mol et al., 2011; Lucas & Pearson, 2012; Rockey et al., 2020; Kjaer et al., 2021). Very few studies address the disadvantages identified by patients (Walters et al., 2003; Heathcote, 2008). In this regard, Walters et al. (2003) mention a certain level of distress associated, for example, with the students’ poor interviewing skills. Heathcote’s study (2008) highlight how marginalization of patient care, strain on the doctor-patient relationship and exposure to potential discomfort or harm may reprensent issues for some participating patients.

That said, the educational contexts in which these studies were conducted entails the capture of a particular reality in terms of the learning environment, pedagogical modalities, and expected learning. Patients’ teaching roles in such clinical contexts tend to remain essentially passive, informal and objectified, as patients mostly engage in transactional relationships, remaining outsiders of the learning encounter (Towle & Godolphin, 2011; McLachlan et al., 2012). Such role also appears to be complicated by the fact that patients primarily visit clinical settings to receive care, not to teach (Towle & Godolphin, 2011). Furthermore, Sehlbach and Rowland (2022) underline that there are many sensible reasons why patients may feel reluctant to participate in learning conversations through their care trajectory given the unequal distribution of risks and rewards between members of the patient-student-educator triad. Considering this, the results of these studies exploring patients’ participation experiences in clinical medical education are only partially applicable to specific non-clinical educational programs or interventions, where patients are expected to play an increasingly important and active role.

We identified a few studies exploring participation experiences in non-clinical interdisciplinary health science education where patients were invited to share their stories and develop teaching techniques based on their past experiences with illness and the health care system (Meehan & Glover, 2007; Lauckner et al., 2012; Romme et al., 2021; Kline et al., 2022). Although none of these studies address the factors that motivated patients to participate, they highlight some benefits patients derive from their participation experience. Participating patients in such contexts derive a sense of contribution, meaning and purpose (Meehan & Glover, 2007; Lauckner et al., 2012; Romme et al., 2021; Kline et al., 2022). Also, patients perceive participation as a valuable experience on a personal level, both socially and therapeutically (Lauckner et al., 2012; Romme et al., 2021; Kline et al., 2022). Studies highlight how patients value the social nature of the interactions with students (Lauckner et al., 2012) and peer-patients (Romme et al., 2021). Participation is also identified as an opportunity for self-reflection and empowerment (Lauckner et al., 2012; Romme et al., 2021; Kline et al., 2022) and as a space to deal with their emotional illness and care experience, allowing the construction of a more coherent, less emotionnaly loaded illness narrative (Romme et al., 2021). Romme et al. (2021) also underline the influence of participation on patients’ awareness of the importance of being proactive in articulating clear preferences through their healthcare trajectories and their increased sensitivity to the perspective and reality of their professionals. On the other hand, some studies also mention how participation can also generate a sense of vulnerability arising from sharing stories associated with an heavy social stigma (Meehan & Glover, 2007; Lauckner et al., 2012) and from a feeling that patient’s narrative is often “seen as an ‘add-on’ rather than a valued contribution” (Meehan & Glover, 2007, p. 153).

Although they provide interesting insights into patients’ participation experience, these studies also have some limitations. For instance, many studies are about the participation of patients with specific clinical profiles (e.g., chronically ill patients, or people with mental health disorders), coinciding with specific educational objectives, such as fostering more favorable attitudes towards a given group of patients, understanding those patients’ reality, or gaining clinical insights from them (Massé et al., 2023). This may allow limited access to the participation experience of patients with other care and illness experiences. In particular, we note how the benefits perceived by participating chronically ill patients are very much in line with what literature identifies as the main mental and social implications of chronic conditions (Larsen and Lubkin., 2013). Finally, since they do not specifically focus on medical education, we suspect the results of these studies to be of limited applicability in the field. Indeed, their interdisciplinary focus may well undermine their ability to grasp the cultural (Good, 1994) and professional (Freidson, 1984) particularities of the medical world and the specific issues arising from such particularities regarding patient engagement.

Finally, we identified few studies focussing on patients’ participation experience in non-clinical medical education contexts (Thistlethwaite & Cockayne, 2004; Watts et al., 2015; Ivory et al., 2017; Flood et al., 2018). In these studies, altruistic considerations are the main motivating factor for patient engagement. Patients appear committed to ensuring that the patient voice is heard (Flood et al., 2018), to help students better understand patients’ reality, improve their clinical behavior, and become better doctors for the benefit of future patients (Thistlethwaite & Cockayne, 2004; Watts et al., 2015; Ivory et al., 2017; Flood et al., 2018). Participation also represents an opportunity for these patients to give back (Watts et al., 2015; Ivory et al., 2017). Only one of those studies (Watts et al., 2015) mention the search for personal gain (i.e., a willingness to learn about their condition and to reduce loneliness and isolation) as a motivating factor. The experience is overall described as positive, useful, interesting (Thistlethwaite & Cockayne, 2004; Watts et al., 2015) and highly emotional (Flood et al., 2018). In terms of what patients get out of it, therapeutic gains are studies’ primary focus. Studies show how participating patients derive a sense of usefulness (Thistlethwaite & Cockayne, 2004), gratitude (Ivory et al., 2017) and achievement (Flood et al., 2018). Participation is also seen as a space to reflect on one’s emotional experience and to move forward in one’s healing process (Flood et al., 2018). Otherwise, participation is also considered an opportunity to interact and talk about one’s health journey and issues encountered, resulting in learning and improved future interactions with doctors (Thistlethwaite & Cockayne, 2004; Ivory et al., 2017).

By failing to bring patients’ perspectives and experiences to the fore, medical education research interested in patients’ engagement in non-clinical medical teaching tends to reproduce the paternalistic power relations the patient engagement paradigm opposes (Hahn et al., 2016). Medical education research thus neglects to conceive patients as subjects, holders of valid experiential knowledge that must be uncovered to improve our understanding of the issues surrounding their participation in medical teaching and to optimize this practice. In addition to the scarcity of studies, we notice that the quantitative design privileged in most of them limits our in-depth understanding of the dimensions of the patients’ participation experiences in specific non-clinical medical educational programs or interventions. The only qualitative study identified (Flood et al., 2018) explore the perceptions of patients regarding their experience of teaching about their cancer experience. They authors underline how their results “align closely to the phenomena of post-traumatic growth (…) where positive psychological change occurs after a trauma; in this case cancer” (p. 190). We thus suspect these results to be coloured by the especially traumatic nature of some patients’ experience with cancer and therefore only partially transferable to other types of patient experience. Considering this knowledge gap, we conclude that the field of medical education would benefit from more qualitative studies focussing on patients’ participation experience in specific non-clinical medical educational programs or interventions.

This study thus proposes to document, through a qualitative design, the experience of patient-teachers (i.e., patients involved in medical teaching as holders of specific knowledge based on their experience of illness and the healthcare system) involved in an educational intervention implemented in an undergraduate medical course at Université Laval, Québec, Canada (see Sect. Our educational patient engagement intervention for more information on the intervention). It will focus on: (1) the factors that motivated patients’ engagement in the intervention; and (2) what patients gained from their participation experience in such context. Our study thus provides an opportunity to develop in-depth understanding of what such participation means and entails for patients.

### Our theoretical background

In their recent theoretical systematic review of patient involvement in health and social care education, Bennett-Weston et al. (2023) observed that this field of research lack theoretical and conceptual underpinning. This is supported by our review of the literature, which includes very few studies with such underpinning. Moreover, when there are, the theoretical and conceptual choices are often rooted in a biomedical perspective that have limited potential for understanding patient engagement in medical teaching as a complex, multidimensional human and social situation. In this research, consistent with what the patient engagement paradigm prescribes, we adopt a stance emphasizing the relevance of critical theory to “think differently about how patients can be engaged in the medical education project in ways that are meaningful and non-tokenistic” and that “provide channels of resistance against traditional power asymmetries” (Sharma, 2018, p. 471). Adopting such an approach will shift the focus of this research to “recognise lived experiences as illuminators of social processes”, “explore the ways in which institutional and social power relations have emotional and material consequences for individuals” and thus consider patients as co-creators of knowledge (Sharma, 2018, p. 477). To our knowledge, this perspective has been rarely adopted in the literature concerned with patients’ experiences of participation in medical education. It appears of particular interest as a growing body of literature emphasizes medical schools’ political and ethical responsibility in structuring patient engagement initiatives that respect the needs, preferences, resources (financial, emotional, etc.) and limitations of individuals while avoiding stigmatization, tokenism and essentialization (Towle & Godolphin, 2011; Roebotham et al., 2018; Lefkowitz et al., 2022; Sehlbach & Rowland, 2022).

## Methods

This qualitative descriptive study is part of a larger research project aimed at evaluating an educational patient engagement intervention’s processes and effects. The modalities of the intervention are detailed in the next subsection to ensure the reader has a thorough understanding of the interventional context.

### Our educational patient engagement intervention

In 2019–2020, our team engaged in a research project aimed at developing, implementing, and evaluating an educational intervention actively involving patient-teachers in undergraduate medical education at Université Laval, Québec, Canada. The intervention targeted the mandatory undergraduate course MED-1210 (Physician, Medicine and Society II). This course is offered to more than 200 first-year medical students each Winter session (January to April). Its main objectives are to develop student knowledge, skills and attitudes related to communication and professionalism and to engage them in critical thinking about their role and future medical practice. The course includes three complementary educational modalities: large group sessions, online sessions, and discussion workshops. These workshops take the form of a series of five small group discussion sessions involving 10–12 students and lasting around two hours each. They are moderated by physician-instructors whose mandate is to host the workshops, facilitate exchanges between the participants, teach formal content (particularly in relation to the legal and ethical aspects of medical practice) and assess students’ learning. During these workshops, students are asked to collectively deliberate on legal, ethical, and moral issues that may arise in the context of their medical practice, based on fictitious scenarios presenting clinical cases. The educational intervention consisted of integrating patient-teacher dyads into the small groups. Those patient-teachers were invited to actively participate in the deliberative discussions by representing alternative perspectives rooted in their experiences with illness and the healthcare system. Patients’ contributions to the discussions could take the form of reflective questions or comments, rooted in a lived experience, to enable students to approach the clinical situations from a different angle. The primary goal of the intervention is to support the development of reflexivity in students (i.e., the ability to learn from one’s experience of a given situation by reflecting on what happened and drawing lessons that will be reinvested in future action).

The intervention was developed in fall 2019 based on the results of a literature review (Massé et al., 2023) and the work of a multi-stakeholder steering committee involving patients (n = 4), students (n = 2), the course leader and members of the research team (n = 3). A pilot intervention was originally scheduled to be implemented in the winter of 2020. It was cancelled in the context of the COVID-19 pandemic. In the winter of 2021, the steering committee revised the initial terms of the intervention to adapt to an online format, with the workshops to take place via videoconferencing platforms. Fourteen (14) patients participated in our intervention during the Winter semester of 2021. They were all initially recruited in the first iteration of 2020 and agreed to participate again in 2021. Our patient recruitment criteria were as follows:: (1) having meaningful experience in the healthcare system as a patient or as a caregiver, (or being a representative of a community organization or patient association who can collectivize knowledge and speak for these patients and caregivers); (2) having the ability to step back from one’s experience, critically question it, and be comfortable discussing it; (3) having an interest in supporting the training of future physicians; and (4) having the ability to speak in small groups. Given the specific requirements of our intervention, patients’ literacy levels were also considered for recruitment. In the context of the intervention, patients were asked to read, understand, and reflect on the clinical cases to participate in the deliberative discussions. Recruitment strategies included the involvement of community organizations, patient networks and individual patient champions.

The research team implemented several strategies to support patient engagement in a responsive and sensitive manner. For instance, a non-mandatory welcoming event was organized to foster a sense of community and to allow patients to validate their interest in participating. During this event, patients got to know each other and discussed the primary reasons for their interest in the project. They also shared their concerns and questions about the project and their participation with their peer-patients, the research team and the course leader. In addition, a mandatory three-hour training session was held in 2020, in person. The main objectives of the training were to clarify the patients’ mandate while emphasizing the importance of a kind and caring learning climate and providing patients with practical insights and tools. The training objective for practical insights and tools was addressed using the principles of philosophical dialogue and thinking skills (Richard, 2021). They allowed patients to identify three main “voice triggers” (i.e., signals which, if felt in the context of the workshops, raise the relevance of speaking out): the feeling of judgment, the feeling that there is a misunderstanding, and the feeling of a lack of consideration. For each trigger a definition was provided, and concrete illustrations were given. Each patient received a paper tool suggesting possible formulations to question or comment on the students’ statements during the discussions. For 2021, the 90 min of online training was a revision of the key elements covered in 2020 plus some technological aspects to support online participation. The day before each workshop, non-compulsory peer preparation sessions were scheduled. Those sessions were an opportunity for patients to read the clinical cases together and initiate a group reflection on what these cases evoked from their specific perspectives. To compensate for the online context which did not allow us to set up a physical place for patients to meet before and after the workshops, patients were invited to share with peers what they liked and disliked about each workshop on a virtual bulletin board. Close communication was maintained between patients and the research team throughout the intervention period.

Initiatives have also been put in place to ensure fair recognition of the value of patient engagement in the project. Patients received financial compensation for their participation in the mandatory training session and in each of the workshops. An online closing event was held to thank patients and acknowledge the value of their participation. Faculty governance members participated and expressed their gratitude to the patients for their engagement. Messages of acknowledgement and appreciation from students and physician-instructors were also shared with patients. All participating patients finally received certificates attesting to their participation as patient-teachers.

### Reflexivity

The first author (JM), whose present research is part of the doctoral project, adopted a transparent approach by recording in a logbook potential biases arising from her presuppositions and epistemological orientations. Indeed, she considers this reflexivity important to identify her *a priori*, to be vigilant in the research process and, ultimately, to be able to name her own limits. In particular, the first author – as well as the other authors who are members of the research team (GODA, MCT) – approached the research with an awareness of her position of power over the participating patients as a young, white, educated academic woman with a favorable health status. She thus anchored her interactions with patients in a posture of humility, openness and sensitivity, recognizing the value, relevance but also the highly emotional nature of people’s experiences of illness and the healthcare system. Great attention was thus paid to the development of an authentic and committed relationship with the patients, to level out the power imbalances but also to make the project a rich human experience for all. The first author has thus attempted to maintain, both in the intervention and the research, more of a facilitating role, aiming to bring to light specific patients’ knowledge, their richness, their importance and their relevance for medical education. Such posture also guided our methodological choices, leading researchers to engage in dialogue with patients as co-creators of knowledge, and then to bring the patient voice to the forefront, both in the results, their interpretation and in the research process itself, which will have contributed a unique perspective on the data.

### Data collection strategy

Data collection was based on 10 semi-structured interviews with patient-teachers who participated in five small workshop groups as part of our intervention. The five small workshop groups were selected via a maximum variation purposive sampling strategy (Creswell, 2013). All patients approached agreed to participate. The 60 to 90-minute interviews were all conducted online in French (i.e., the first language of the patients as well as that of the members of the research team) by the first author (JM) between June 18 and July 8, 2021 (that is, between two and three months after the end of the workshops). A preliminary interview guide was constructed based on our literature review (Massé et al., 2023) and our research objectives. The guide was reviewed by a patient-teacher prior to the interviews to ensure that the questions were clear and appropriate and that they were consistent with our research objectives. The final guide incorporated feedback from the patient-teacher. The interviews were recorded and transcribed for analysis. NVivo software was used.

### Data analysis

A thematic analysis (Braun & Clarke, 2006) of patients’ motivations for participating was conducted using a primarily iterative and inductive approach. Regarding what patients have gained from their participation experience, the thematic analysis used the Kirkpatrick framework *a posteriori* to classify preliminary codes. In the context of this study, we applied the original version of the Kirkpatrick framework, which has four levels of educational intervention outcomes: Level 1 – Reaction (i.e., learners’ satisfaction, appreciation); Level 2 – Learning (i.e., changes in attitudes and perceptions, acquisition of new knowledge and skills); Level 3 – Behaviors; and Level 4 – Results. This framework is widely applied in education and is used to categorize the effect of a given educational intervention on learners (Boet et al., 2012). We deemed it relevant as it helped go beyond what was formally gained (i.e., attitudes, knowledge, skills and behaviours) and left us room for a deeper critical questioning of participants’ reactions and appreciation of their experience while interrogating their emotions and feelings. Critical theory has been mobilized in a transversal way to guide our view of the data.

The first author (JM) was the main responsible for the analytical process. Different strategies were put in place to ensure quality and rigor criteria were met (Tracy, 2010; Creswell, 2013). In particular, the fourth author (GODA) was involved in a peer review process of the preliminary analysis grid. Her input ensured internal homogeneity and external heterogeneity of the themes (Braun & Clarke, 2006). The consequent revision of the grid was discussed until a consensus was reached. Subsequently, a two-hour co-interpretation meeting was conducted with patient-teachers who participated in the intervention. During this meeting, the first author (JM) shared a preliminary coding grid with patients and welcomed constructive feedback in an atmosphere of humility and openness. Patients’ feedback made it possible to verify if the preliminary results painted an accurate picture of their motivations for participating and what they gained from their experience. This reflective and collaborative approach was intended to shed new light on the research and to foster deeper and richer analyses (Tracy, 2010; Creswell, 2013). The involvement of two patients (SG and LV) in the preparation of this article had similar objectives: to make sure patient-teachers’ voices are at the core of this paper and that it accurately reflects what is important to patients. This practice has therefore contributed to the further validation of the data.

### Patients’ contribution as co-authors

The two patient co-authors were involved in the intervention as patient-teachers. They were chosen as co-authors based on their interest and their acknowledged ability to take a critical look at the data, to offer a fine analysis and to collectivize participation experiences. Patient participation in the preparation of the manuscript was carried out in two stages, which we have jointly agreed upon beforehand. First, the first author (JM) produced a detailed summary of the draft manuscript which she submitted to the SG and LV. To avoid the language barrier, this summary has been produced in French. Patients were asked to critically review the summary by noting all questions, misunderstandings, disagreements, inaccuracies, complementary ideas and all other comments. Here is a sample of the questions that were submitted to the patient co-authors to support them in the review process:

- In the background section: does the chosen angle seem interesting to you? If not, do you have any suggestions for adjusting it so that it better reflects your vision of the issue we are addressing?
- In the results section: does the interpretation of the results sound right to you? Are there some places where it’s going too far, where it’s not going far enough, where you think it’s misunderstanding what the patients meant?
- In the discussion section: do the points brought out seem relevant to you? Are there any others that you think should be discussed?
- Throughout the text, do the words used, the formulations, the tone seem appropriate to you?
- Are there passages or sentences that you particularly appreciate, which seem particularly interesting to you, and on which the emphasis should be put when writing the final article?
- Do certain passages raise questions or lack clarity?
- Do certain passages just bother you (even if you don’t really know why)?

Following their review work, both patients transmitted their notes to JM. Certain points were discussed when required, to ensure a common understanding of the comments and to remove misunderstandings on both sides. The first author formulated a draft manuscript incorporating the feedback received. Before submission, she gave a thorough presentation of the final text to patient co-authors during a 90-minute meeting. It was an opportunity for the patients to confirm that their comments had been considered correctly and to ask for some final adjustments. Both patients received a financial compensation lump-sum for their participation in the preparation of the article.

## Findings

### Participant profile

The participants in this study represent a variety of demographic, health, and socioeconomic profiles as shown in Table 1.

**Table 1:** Study participants’ profiles

**Gender**	Women	6
	Men	4
	*Total*	*10*
**Health profile**	Chronic disease with physical and/or cognitive manifestations	1
	Chronic disease with no physical or cognitive manifestations	5
	Physical disability	2
	Mental Health issues	2
	*Total*	*10*
**Socio-economic profile**	Living in poverty or receiving social assistance	3
	Employed	3
	Retired	4
	*Total*	*10*

In addition to their own health issues, many participants had recently been family-caregivers at the time of the research. Some participants also had an history of involvement as peer-helpers in community care settings (n = 2), as patient-partners in research or in care setting interventions (n = 4) or as standardized patients (n = 1). All participants also had some history of commitment in community organizations or social causes.

### What motivated patients to participate

The factors that motivated the patient-teachers to participate in our intervention, as identified retrospectively by our participants, fall into two main categories: (1) motivations arising from perceived consistency between who they are and the characteristics of the project in which participation takes place, and (2) motivations arising from conceiving the project as a means to reach goals at the individual and social levels. Table 2 summarizes the main themes and sub-themes that emerged from the analysis.

**Table 2:** Summary of the motivations for patients’ participation in an undergraduate medical education intervention

Themes	Sub-themes
**Patients’ perception of consistency between themselves and the project in which the participation takes place**	• Perceived consistency between the foundations of the project and one’s own convictions and beliefs • Perceived continuity between the context of participation and one’s personal history
**Patients’ identification of the project as a means to achieve personal and social goals**	• Participation to fulfill some personal needs • Participation to contribute to social change

#### Perceived consistency between self and the project

*A perception of consistency between the foundations of the project and one’s own convictions and beliefs*.

For many patients, participation was motivated by the finding that the project and its foundations, objectives and practices were in line with their personal convictions and beliefs. It included, for instance, a belief in the value and relevance of meaningful patient involvement in healthcare and, more specifically, in the education of healthcare professionals, as mentioned here:

*For me, it would make complete sense to say, ‘the central people in the care situation are the people we care for’. It’s essential that those people be involved [in health sciences education] in some way. (Patient 1)*

Similarly, few patients emphasized that they were motivated by a perceived coherence between the deliberative process proposed in the workshops and their conviction as to the interest and benefits of co-construction and collective reflection, especially regarding the opening of one’s horizons:

*That was the aspect that interested me a lot: the community aspect. We think together, we learn to consider aspects that we might not be inclined to consider. It allows us to have a more global vision. (Patient 8)*

*A perception of continuity between the context of participation and one’s personal history*.

Participants also noted how our intervention fitted harmoniously in their life trajectory. For some, the project was consistent with a long-standing interest in the medical field or, more broadly, for caregiving and health. Others were motivated by the finding of a coherence with past experiences in the professional field (in human resources, for example) or of social engagement, as highlighted here:

*I have always been socially engaged. So, for me, “patient-citizen-partner”, it fits exactly in this line. (…) For me, this commitment [within the intervention] is just a continuity. (Patient 2)*

In short, these results suggest that patients were willing to engage as teachers in the context of our intervention when they found our project made sense for them and was coherent with their life trajectory.

#### The project as a means to reach personal and social goals

*Participation to fulfill some personal needs*.

Patient participation in our intervention was also motivated by a perception of the project as a means of meeting some individual needs. For example, some patients mentioned participating to satisfy a need to connect with peers and thus belong to a community of people sharing a unique experience with illness and the healthcare system:

*You talk about your health trajectory and then you have the feeling that people you talk to have experienced the same kind of things. (Patient 10)*

Some patients also said they conceived participation as a means of meeting a need to feel useful and that they make the most of their experiential knowledge, despite the limitations caused by their illness. This feeling of usefulness even became, for some of these participants, a rich source of personal satisfaction and thus contributed to giving meaning to their lives. This is illustrated here by the words of a patient living with mental health issues:

*That’s something that turns me on, and if I have moments of pleasure left in life, that’s it: it’s the feeling of usefulness that I can feel. (Patient 9)*

Some said they saw participation as a way to overcome frustrations associated with challenging past experiences in the healthcare system. Participation in the project thus became a means of initiating a constructive and positive shift, as underlined here:

*I accumulated frustration, and I lack trust in the healthcare staff (…). I need to move on from all of this. (Patient 7)*

Likewise, participation was also sometimes conceived as a means of developing an in-depth understanding of the factors influencing certain medical behaviors, thus gaining new tools to critically assess one’s own care experience:

*I wanted to understand the [decision-making] pathway doctors follow. And why they come to… It’s because… I have a resident as a family doctor. I can’t get used to him. What annoys me the most is that he is on the computer all the time. You want to say something, he stops you. He must finish writing. (Patient 3)*

These results highlight that patients were motivated to participate by their perception of the project as a means of satisfying their need to develop significant social relationships with peers, to feel useful, to move forward, to rebuild their confidence in the healthcare system and its actors, and to better understand their own care experience.

*Participation to contribute to social change*.

Beyond the individual level, the data also suggest that participation was motivated, for many patients, by a desire to contribute to the transformation of health practices, structures, and policies. In particular, some were motivated to participate by negative experiential findings drawn from the experience of inappropriate approaches, methods and discourses in the healthcare system, as illustrated here by the words of a patient experiencing mental health issues:

*I have experienced restraint [i.e., the action of impeding a person’s mobility (by physical or chemical means)]*, *isolation. I don’t understand. I can’t believe that men and women of science use punishment to heal people. (Patient 6)*

Participation was perceived, in these cases, as a means to co-construct knowledge that has the potential to bring about change while preventing the perpetuation of these problematic approaches, methods and discourses that have deleterious emotional, physical or material effects on patients and their families.

On the other hand, positive experiential findings also motivated participation from an altruistic perspective. For patients satisfied with their care experience, participation represented a means to express gratitude by contributing their experiential knowledge, as the following quote highlights:

*For me, this is very important: giving back to the healthcare system what the healthcare system gave me. It’s paying forward. (Patient 10)*

In brief, these results highlight how patients are driven by a desire to contribute their specific knowledge to the transformation of healthcare practices, structures and policies, given their either positive or negative past experiences within the healthcare system.

### What patients gained from their participation experience

The presentation of results regarding what patients gained from their participation experience is inspired by Kirkpatrick’s framework. The results mainly refer to the framework’s level 1 (reaction: learner’s appreciation, satisfaction) and level 2 (learning: change in the learner’s attitudes, knowledge, skills). Table 3 provides a summary of the main results of this section.

**Table 3:** Summary of what patients gained from their participation in an undergraduate medical education intervention

Themes	Sub-themes
**Patients’ appreciation of the participation experience**	• A positive, enriching, motivating yet uncomfortable and destabilizing experience • An experience arousing emotions and feelings (e.g., feelings of gratefulness, valuation and appreciation; feelings of frustration, dissatisfaction, stress and insecurity) • An opportunity to be known and to assert one’s beliefs and opinion, to be exposed to other perspectives and to create meaningful social bonds
**Changes in patients’ attitudes toward medical education, healthcare professionals and themselves**	• A deconstruction of patients’ biases against medicine, medical education and medical students • The development of critical thinking about one’s experience of care and the conditions of medical practice • An increased confidence in the relevance and value of one’s experiential knowledge and in the future of the healthcare system
**Development of new understandings, knowledge, and skills**	• A better understanding of the thinking mechanisms underlying medical practice • New knowledge about patients’ rights and some clinical notions and issues • Leverage impacting patients’ ways of interacting with the healthcare system and other spheres of their lifes

#### Patients’ appreciation of the participation experience

All patients perceived participation as a positive, enriching, and motivating experience, though some found it to be inherently uncomfortable and destabilizing:

*It’s like when you go into the woods with people you don’t know or you’re trapped in an elevator. You don’t know how it’s going to turn out. These are always great adventures. (Patient 9)*

*An experience arousing emotions and feelings*.

More specifically, patients reported feeling grateful, valued, and appreciated as a result of their participation. Some associated these feelings with the whole project’s commitment to advocating for the fair recognition of patients’ experiential knowledge in medical education as illustrated here by the words of a patient speaking directly to the first author:

*The fact that you’re doing this… yes [as a research project] it has a specific purpose but in terms of humanity, it is to believe in us. It’s gratitude that I have for that. (Patient 7)*

Gratitude to the research team was also expressed by some patients in reference to the support offered for participation, as mentioned here:

*I felt pampered throughout this process, always made sure of my understanding and my agreement and my needs, frequently. Yes, that’s it… I’m going through the motions of a baby being rocked but I really felt very considered in all of this. It sure feels good. (Patient 1)*

Others associated these feelings of gratitude, valuation and appreciation with their concrete experience of the workshops. Indeed, some patients mentioned how they felt welcomed and listened to with openness and interest in an atmosphere of mutual respect. This quote shows how these feelings were, for some patients, accompanied by a certain sense of surprise at the discrepancy between the workshop experience and the expected power dynamics between patients and medical students:

*The students could react and respond to what we were saying. You thought they are the ones who are supposed to know more, but no, they discussed with us, they considered us as people in our own right. (Patient 7)*

Other patients felt grateful to the other patient-teacher of the dyad for the quality of one’s presence and words during the workshops, that was source of support, confidence, and inspiration:

*The presence with a capital P of my partner, in all that he gave. It was magnificent to see him go in his eloquence, his way of verbalizing things, the conciseness that he has, of using the exact terms, in a language completely adapted - I consider in any case - to the students, to the situation. (Patient 1)*

That said, this sense of appreciation and consideration appeared affected by the learning climate that developed differently within each group. For instance, a task-oriented learning climate had led some patients to feel that formal content took priority over patients’ specific input:

*They [the students] listened to us with interest, I have no doubt about that, but I felt that there was a subject to study, and then there was an exam. In Workshop 5, when they had to turn in their analysis of a case for evaluation… I never heard anything that was related to what we [patients] had been given as information or our experience. (Patient 4)*

Some patients also mentioned that they felt some dissatisfaction and frustration along the way. For instance, they associated these feelings with (1) a divergence in posture with the other patient in the dyad or (2) the instructor-patient power dynamic perceived in some groups:

*I felt like I had to be a little careful about what I said. Because of what [the instructor] gives off, it doesn’t make you want to argue. (…) I quickly realized that [the instructor] had me… I’m here, you’re here [miming with an arm one level up and then one level down]. She gave us [the patient-teachers] a place but… a place. Not the place I would have liked to have. (Patient 4)*

Dissatisfaction and frustration were also associated for some with a perceived discrepancy between the lessons put forward in the workshops, including the importance of a patient-centered approach that supports a healthy, collaborative and effective care relationship and their real-life, more paternalistic and procedural experience of care, as illustrated here:

*While I was a patient-teacher, I also went to doctors for treatment. There was a huge clash (…).[During the workshops] I felt like I was in a Walt Disney movie. I found it difficult. (Patient 6)*

Some patients also mentioned how participation generated a certain level of stress and insecurity. For instance, this was associated with: (1) doubts about one’s own literacy level and ability to effectively read and understand the paper-based clinical cases, (2) the perceived role of the project in leveraging the expansion of patient-partnerships at the institutional level, or (3) the perceived complexity of the patients’ mandate at the interface between the rational and the experiential:

*One of the things I found difficult was the patients’ stance within the intervention. The fact that we had to refer to an analytical grid [referring to the paper tool provided to patients during the mandatory training] to reflect to students what they said while also sharing our personal experience. I found this extremely difficult to reconcile. That made me feel uncomfortable. I wasn’t sure how to do what was expected. (Patient 9)*

*An opportunity to be known and to assert one’s beliefs and opinion, to be exposed to other perspectives and to create social bonds*.

Retrospectively, participants also identified the participation experience as an opportunity to make themselves known and to assert their opinions and convictions regarding, for example, issues of access to care for certain groups and communities. It was also an opportunity to be exposed to other experiences, postures and ways of thinking through interactions with other patients, physician-instructors and students.

Finally, participation was, for some, an opportunity to create an authentic encounter and meaningful quasi-familial ties with the students and to create a sense of belonging to the student group, as expressed here:

*When it came time for me to sit at the screen, I was: ‘I’m going to join my gang, I’m going to see their faces, I’m going to join so-and-so.’ (…) It was as if I knew them from before. (Patient 8)*

However, according to patients, in some groups, the experience of such an authentic encounter with students was hindered by students’ unreadiness to engage (e.g., students’ level of fatigue, their turbulent work environment), the facilitation modalities (e.g., some instructors chose to divide the small group in half for more intimate deliberation sessions and then come back to the whole group for key messages, while other instructors preferred to conduct whole group deliberations) or the professional distance maintained between patients and students. Often, being online, with little room for informal exchanges, also limited the development of a sense of connectedness.

#### Changes in patients’ attitudes toward medical education, healthcare professionals and themselves

*Deconstructing biases against medical education and medical students*.

The participation experience allowed patients to initiate a deconstruction of their own prejudices towards medical education and the teachings it conveys. In this regard, participants found out that the patient was much more considered and valued throughout the curriculum than they initially thought. Similarly, the experience challenged some preconceptions about the deeper motivations for choosing a medical career, which were now perceived as fundamentally altruistic:

*I saw that people who went into medicine were not going there to get rich. (…) It’s really, viscerally, to help people. (…) I had huge negative preconceptions. (Patient 6)*

Participation was also an opportunity to see medical students in a different light and to be surprised by their interest in acquiring non-technical skills (such as those covered in the course targeted by our intervention), by their openness and good understanding of patients’ needs, and by their demanding learning reality. This new perception has led to increased confidence in future physicians’ ability to become ‘good doctors’ and to the development of a more understanding and conciliatory attitude towards them:

*They are young people. They want to learn. They begin and then they find themselves at 26–27 years old in charge of patients. A lot is expected of them. (Patient 2)*

*Developing critical thinking about one’s experience of care and the conditions of medical practice*.

Participation in our intervention also allowed some patients to critically reflect on their own care experience. Indeed, they were often tempted to question such experience in light of the discussions and lessons learned during the workshops, as illustrated here:

*I told students a few stories about my personal healthcare experience. [The instructor] asked them how they would have reacted in such situations. I listened to their answers, and I thought: how could [my doctor] not be able to answer me like that? (…) It surprised me! (Patient 5)*

Moreover, the experience fostered critical thinking about the impacts of medical practice conditions on patients’ health trajectories. This point is illustrated by the following quote from a patient who, considering her own experience, was specifically concerned about the role played by medical practice conditions in maintaining access-to-care inequities:

*I know they have a code of ethics that they must follow. Maybe in this code, there should be some changes. This is what I felt throughout the workshops. (Patient 5)*

*Regaining confidence in the relevance and value of one’s experiential knowledge and in the future of the healthcare system*.

Most patients believed they had made an unprecedented contribution to the learning of future physicians by sharing their experiential knowledge or that they had “planted some seeds” (*Patient 9*). However, most of them explicitly mentioned that the online context affected this sense of usefulness because of, for instance, the minimal non-verbal and informal feedback patients received from students, as expressed here:

*That remoteness, it made it hard for me to grasp how they receive what I shared. Does it tell them anything? Does it make any difference to them? (Patient 1)*

Despite that, participation led some patients to regain confidence in themselves and their ability to make a meaningful and useful contribution to medical education, as highlighted by a patient with lower literacy levels:

*Sometimes I thought: ‘Maybe I’m asking a silly question’. But in the end, it wasn’t that silly because it led students to say, ‘Oh yes, you’re right, I should go further, I should think about it more.’ (Patient 5)*

Finally, some patients also highlighted a renewed confidence in the future of the healthcare system and its ability to better integrate certain considerations that are crucial to patients, as expressed here:

*There are a few things that are going to change, and that makes a big difference for me to know that, to think that, to believe that. (Patient 7)*

Beyond appreciation, these results highlight how participation in the context of our project enabled patients to initiate positive attitudinal changes and critical thinking.

#### Development of new knowledge and skills

Lastly, experiencing the deliberative process within the workshops allowed some patients to better understand the thinking mechanisms that underlie medical practice. Patients also reported gaining new knowledge about patients’ rights and clinical issues, as illustrated here:

*I know now that the restraints go beyond what I had in mind, with the arms. (Patient 7)*

These new understandings and knowledge were identified by some patients as a source of leverage that could impact their ways of interacting with the healthcare system either through improving their health literacy or through a sense of empowerment allowing them to become reinvested in the management of their own care:

*I will have even more power over my future treatments with my doctor. There are things that I understand more, and I will be able to ask more specific questions. (Patient 4)*

This empowering nature of learning is also reflected in the desire of some patients to reinvest it in other spheres of their lives, in community and associative involvement for example:

*I loved it and it led me to run for the board of directors of the [association]. It gave me even more confidence. (Patient 4)*

These results highlight the acquisition of new knowledge, understanding and skills that can be mobilized by patients in their professional, personal or care pathways.

## Discussion

This study provides an in-depth and multidimensional picture of the factors that motivated patients to participate in an undergraduate non-clinical medical education intervention requiring their active and meaningful contribution to a small group deliberative discussions based on their lived experience with illness and the health care system. It also provides an in-depth understanding of what patients gained from their experience of participating in such context. In thus contributes important knowledge to the medical education field by opening up a new critical perspective on the understanding of the patient-teacher concept in medical education and its practical implications for medical schools.

### Patients engagement through the lens of critical theory

*A posteriori*, the analysis of the results from the perspective of critical theory allows us to establish links with the conceptual basis of Paulo Freire’s critical pedagogy which has influenced people working in education, community development, community health and many other fields (Freire Institute, 2023). Positing education as a political enterprise, Freire’s educational approach establish a critical relationship between issues observed and experienced in the world, knowledge and positive action for social change (Freire Institute, 2023). While the oppressor-oppressed dynamic and the idea of liberation through social action are at the basis of Freire’s work (Freire, 2015), we postulate here the patients as the less powered party within the health sector and, more specifically, in medical education. This assumption is based on the argument made by other authors that the traditional social dynamics in medical education, rooted in the long-lasting culture of a health system that has fundamentally built itself on scientific knowledge (Dumez & Pomey, 2019), “led to and perpetuated the types of power imbalances that excluded and alienated patients” (Bleakley, Bligh and Browne, 2011, p. xiii).

#### Patients engaged as active teachers and learners

For Freire (2015), no educational process can claim to be neutral. He contrasts a “banking” education model in which knowledge is conceived as a gift handed down from those who consider themselves knowledgeable to those they judge ignorant, to a dialogical education based on the authentic encounter of human beings. Our results suggest that patients are engaged in the participation experience as active teachers and learners, thus overcoming the internalized feeling of powerlessness associated with their historical role as determined objects (Freire, 2015; Aujoulat, 2007) or as passive consumers of health knowledge (Ko, 2016). Indeed, the results reveal patients as thinking and knowing subjects who feel motivated to participate as a result of a reflective exercise through which they put participation into perspective with their own historicity, their own beliefs, their own understanding of the world. They also adopt an active and reflective learner’s posture, being motivated to participate by the idea of the dialogical collectivization of knowledge and experiences to understand the world in all its complexity. Such learner’s posture is also reflected in what patients gain from their experience. In particular, when they mention a deconstruction of their own prejudices towards the medical field, a critical reflection on their own care experience, and a better grasp of the scope and relevance of their experiential knowledge, patients show how they can enact their self-normative potential (Barrier, 2012, 2014) through constant confrontation of their specific knowledge with biomedical and professional knowledge, to ultimately extend the learning efforts they have initiated about their experience. This is very much in line with the idea of conscientization of the oppressed which constitutes, in Freire’s perspective, “the process of developing a critical awareness of one’s social reality through reflection and action” (Freire Institute, 2023b). Following authors such as Bleakley and Bligh (2008) or Rees et al. (2007), such a conception of patient-teachers as active learners appears essential to the co-production of knowledge in a perspective of dynamic and synergistic mutuality where all actors are epistemologically curious and engage in a dialogue to learn from each other.

The non-neutral posture of patient engagement in medical education also translates into patients’ conception of participation as a means of meeting individual needs. In particular, what emerges from our results is that participation was conceived as a means to fulfill a need to engage in a positive and constructive process. Such a process was aimed at enabling patients to feel a sense of accomplishment and move forward as they were seeking an overall improvement in their well-being, self-esteem and satisfaction. This highlights how participation is part of a life path that is often marked by vulnerability and oppression, which has taken an emotional toll on patients. More than a simple search for personal therapeutical gains, the expectation of meeting individual needs here highlights the meaningfulness that patients attach to participation and its potential impact on their journey.

#### Patient engagement as emancipation

Our study highlights altruistic motivations for patient participation, which underscore patients’ concerns that go beyond one’s individual situation. Studies concerned with patient participation in either clinical or non-clinical medical education contexts also highlighted how such altruistic considerations are central to patients’ engagement. For instance, studies highlight that patients participate to help students become better doctors for the benefit of future patients (Thistlethwaite & Cockayne, 2004; Watts et al., 2015; Ivory et al., 2017; Flood et al., 2018) or to give back (Watts et al., 2015; Ivory et al., 2017). Looking at these considerations from a critical angle however gives them a whole new transformative and emancipatory dimension while highlighting participation as a mean for patients to deal critically with reality and ultimately discover how to participate in the transformation of their world. Freire’ work (2015) underlines how the process of conscientization must allow such emancipation of the oppressed so that they get to transform their social reality. That’s where it connects knowledge to action. In the context of our study, this perspective emphasizes patients’ willingness to engage in the co-construction of transformative knowledge towards a revision of power dynamics in health and to consequently constitute themselves as actors of social change. Furthermore, in this regard, our results emphasize how socially engaged patients (see Sect. Participant profile for more information on participating patients’ history of social engagement) carry a voice that extends beyond their individual situation, which brings a collective and political perspective based in the broader socio-economic context in which those patients live.

Our results also show how patients identify some of the learning gained from the experience as enabling transformation in the way they interact with the healthcare system and, more broadly, in their personal and professional lives. The empowering and capacitating nature of this learning is emphasized as it appears likely to equip people to regain control over their live, their healthcare and to achieve some of their ambitions. It thus suggests that beyond an isolated understanding of participation as transformative action in itself, our results allow to see longer-term effects on patients’ willingness and ability to engage in social change.

### Practical implications for medical schools

By bringing patients’ perspective to the fore, our study presents a critique of medical education practices that conceive of patients’ input as “interesting teaching ‘material’, often no more than a medium through which the teacher teaches” (Spencer et al., 2000, p. 851). The formative character of some of those practices (e.g., unidirectional patient testimonials) is undeniably relevant to students since they contribute to the contextualization of one patient’s case and, in doing so, provides better understanding of the issues raised and fosters the acquisition of the skills required to properly address it (Lechopier & Granier, 2017). Patient testimonials, rooted in a tangible experience of illness and the healthcare system, also have the capacity to arouse students’ emotions, thus fostering some key learnings through the critical inquity of one’s values and cherished beliefs (Greene & Boler, 1999; Tremblay et al., 2021). However, they do not restore the patient’s role as co-constructor of knowledge. Our study thus calls for a transformation of institutional approaches to patient engagement from a utilitarian perspective to a more humane and critical one, where patients are conceived as human beings in their own right, capable of engaging in a dialogue of win-win teaching and learning. Indeed, the better understanding of patients’ motivations and what they gain from their experience engages the responsibility of institutions to put forward patient engagement initiatives using a sensitive approach based on a deep and holistic understanding of what such participation means and entails for these people. Authors caution health educators and practitioners that patients’ singular experiences are always somehow at risk of being assimilated into an objectified, dehumanized, and universalized patient perspective (Rowland et al., 2017; Sharma, 2018) and instrumentalized in the reproduction of pervasive power imbalances rather than being used to resist or transform them (Rowland et al., 2017). Freire (2015) consistently calls for such educators’ constant reflective re-examination of their practice for its authentical commitment to people.

Our use of a qualitative design allowed for a particularly detailed and thorough understanding of elements in the context of our intervention that impacted what patients got out of their experience and that were into the instructors’ and educators’ control zone, for instance: the learning format (e.g., online/in person), the learning climate within the workshops (e.g., professional/friendly), the facilitating methods (e.g., directive/democratic) and the assessment methods. Also, through the informal and hidden curriculums (Hafferty & O’Donnell, 2014), instructors had the power to influence students’ perception of what is to be valued in a course and, consequently, the relevance and importance of patients’ specific knowledge. This suggests that special attention should be given to training instructors and educators to develop a common understanding of (1) what participation means and entails for patients, (2) what role and responsibility should be given to patients (see Gross et al. (2017) for a concrete example of patients as real co-teachers with extended responsibilities in terms of teaching the patient perspective and assessing students’ learning), and (3) the concrete implications for their own teaching practice. There was no space for such training within our intervention.

Our study also highlights the importance of implementing initiatives aimed at supporting patient participation, to enable the collectivization of their experiences and to foster peer-support and psychological safety. Indeed, by highlighting the importance for patients of developing meaningful social relationships and the emotional nature of the experience, our results underscore the relevance of initiating and facilitating a supportive peer community for patients. It is however a call for caution regarding training as a space of power for educational programs and interventions leaders. There are several schools of thought regarding the prior training of patients as a best practice. We have chosen, in the context of our intervention, to offer such training to patients (see Sect. Our educational patient engagement intervention for further details). Although this training was designed to ensure that patients clearly understand their mandate and to develop their sense of competence, our results highlight how such training runs the risk of creating insecurity by imposing a posture or by orienting the narrative in a way patients are not all comfortable with. This raises the relevance of training approaches such as that adopted by Gross et al. (2017) who have chosen to rely on the principles of reflective practice, allowing the emergence, over time, of a progressive formalization of patients’ way to intervene and the content they address. Consequently, Gross et al. (2017) didn’t rely on an initial training for patients but on a co-developed program, based on the training needs felt and expressed by the patient-teachers’ peer community along the way.

### Limitations and avenues for future research

The results of this study should be interpreted while taking their limitations into account. In particular, the study presents the perspectives of only 10 participants. While consensual themes emerged from the analysis, other relevant examples and nuances could potentially have emerged from more interviews. In particular, despite efforts to recruit patients from a variety of backgrounds and life histories, our participant group was very culturally homogeneous. We assume that the contribution of immigrants, racialized or Indigenous people could have opened up new horizons in this research. Accordingly, we argue that patient engagement initiatives should be adequately supported to ensure that recruitment strategies are tailored to culturally diverse populations and groups. That said, recruiting people from such populations and groups requires particular attention as it implies designing interventions that are culturally safe. We also acknowledge that having patients with a history of social engagement may have had an impact on the reasons outlined for participating in our intervention.

Still, the steps taken to ensure research quality and rigor allowed us to validate that what is presented here resonates well with the patient experience in the context of our intervention. Moreover, the range of perspectives represented in this study allows us to highlight different views on participation, including positive, negative, and nuanced views, which may make our results applicable to similar educational contexts.

Future research may aim to document patient experiences in the context of larger studies involving more patients and allowing the detection of variability by gender, socioeconomic status, health status, etc. We were not able to detect these variabilities in the context of this study.

## Conclusion

This study provides an opportunity to better understand, from the patient’s perspective, what motivates patients to participate in medical teaching and what they gain from such experience. It puts patients front and center to address the power issues that have traditionally contributed to the exclusion and alienation of their experience and specific knowledge in medical education research (Bleakley et al., 2011; Sharma, 2018). The results support the promotion of a critical and emancipatory view of the patient-teacher and of renewed interventional approaches that value patients’ experiential knowledge as complementary to medical knowledge in teaching and learning the Art of Medicine.

## Data Availability

In accordance with our ethical commitments to participants, the datasets generated during and analysed during the current study are not publicly available. However, they may be available from the corresponding author on reasonable request and with permission of the participants.
